# Evaluating the Reliability of a Microperimetry-Based Method for Assessing Visual Function in the Junctional Zone of Geographic Atrophy Lesions

**DOI:** 10.21203/rs.3.rs-5183845/v1

**Published:** 2024-12-23

**Authors:** A. Yasin Alibhai, Eric Moult, Muhammad Usman Jamil, Khadija Raza, Marco U. Morales, Ramiro Ribiero, Caroline R. Baumal, James G. Fujimoto, Nadia K. Waheed

**Affiliations:** Boston Image Reading Center; Massachusetts Institute of Technology; New England Eye Center, Tufts Medical Center; New England Eye Center, Tufts Medical Center; Apellis Pharmaceuticals (United States); Apellis Pharmaceuticals (United States); New England Eye Center, Tufts Medical Center; Massachusetts Institute of Technology; New England Eye Center, Tufts Medical Center

**Keywords:** microperimetry, geographic atrophy, fundus auto fluorescence, age-related macular degeneration, scotomatous points

## Abstract

**Purpose:**

To assess the repeatability of a microperimetry methodology for quantifying visual function changes in the junctional zone of eyes with geographic atrophy (GA) in the clinical trial context.

**Methods:**

A post hoc analysis of the OAKS phase III trial was conducted, which enrolled patients with GA secondary to age-related macular degeneration. Microperimetry using a standard 10–2 fovea centered grid was performed at baseline and follow-up visits. GA regions were traced on fundus autofluorescence (FAF) images. Two graders independently registered baseline microperimetry images with baseline FAF images in a sampling of 30 eyes from the OAKS study. Agreement between the two graders’ assessments of mean sensitivity and the number of scotomatous points within a ±250 *μ*m GA junctional zone was assessed.

**Results:**

The intraclass correlation (ICC) and coefficient of repeatability (CoR) for the mean junctional zone sensitivity were 0.994 and 0.349 dB, respectively. The ICC and CoR for the total number of scotomatous points within the junctional zone were 0.997 and 0.218, respectively.

**Conclusions:**

The repeatability of the methodology and its compatibility with standard MP acquisitions appear to make it well-suited for identifying and analyzing retinal sensitivity within high-risk areas of the retina.

## Summary Statement

We present a microperimetry-based methodology for assessing visual function changes in the junctional zone of geographic atrophy lesions using a standard 10–2 fovea centered grid in a clinical trial context. The approach’s repeatability and compatibility with standard microperimetry grids may make it useful for assessing the effects of GA therapeutics.

## Introduction

Geographic atrophy (GA), the late stage of non-exudative age-related macular degeneration, significantly reduces quality of life^1,2^. The consequences of visual impairment in GA patients include an increased risk of falls, difficulty reading, driving, and recognizing faces, and ultimately, the loss of independence^3,4^. In 2023, two complement inhibitors were approved by the Food and Drug Administration (FDA), becoming the first approved therapies for treating GA. These therapies were shown to significantly slow the enlargement of GA as measured on fundus autofluorescence (FAF). However, for these and future GA therapeutics, it is important to understand their effects on measures of visual function in addition to structural measures.

Sensitive and reliable measurement of visual function in GA patients remains a challenge. The standard assessment of visual acuity is typically performed using eye charts, which is a reliable and reproducible test when patients have a healthy macula and good fixation. In patients with extrafoveal lesions, there can be significant GA growth without any effect on visual acuity, even with patients complaining of worsening visual function. Similarly, once the central fovea is involved, there can be further growth of the GA lesion without additional changes in visual acuity—in this situation, too, the patient’s functional capacity may decline as their scotoma enlarges. Therefore, it is important to develop approaches that can assess the visual function changes that accompany the structural changes that occur as GA lesions enlarge.

Microperimetry (MP) provides a functional mapping of the retina that is precisely correlated to fundus anatomy. Because MP assesses light sensitivity at specific, predefined retinal loci that can be longitudinally tracked, MP may be a sensitive measure of visual function changes in patients with GA^5,6^. Nevertheless, conventional MP analyses (e.g., mean sensitivity across all stimulus points) are limited in that a substantial proportion of stimuli may fall within the region of atrophy or be located far from the atrophic region over a still healthy retina area, particularly when large sparse grid distribution are selected, and are, therefore, unlikely to change as the GA lesion enlarges. This decoupling may lead to an underestimation of therapeutic effects^7^. Thus, developing and evaluating approaches that analyze MP sensitivities within a junctional zone of the GA lesion may lead to more sensitive measurements of the effects that GA therapeutics have on visual function^5,6^. The aim of this study is to assess the repeatability of an MP methodology for quantifying visual function changes in GA junctional zones in a clinical trial context.

## Methods

### Study Design

The repeatability of our MP analysis workflow was evaluated on a cohort of GA patients from the OAKS study (NCT03525600)^8^. The OAKS study was a 24-month, multicenter, randomized, double-masked, sham-controlled, phase 3 study, which enrolled patients at 110 clinical sites. The study adhered to protocols approved by the institutional review board of each site and complied with the Declaration of Helsinki. The inclusion and exclusion criteria of the OAKS study are described elsewhere^8^. Patients were randomly assigned (2:2:1:1) by a central web-based randomization system to intravitreal 15 mg per 0.1 mL pegcetacoplan or sham either monthly or every other month.

### MP Testing

MP testing was conducted using the Macular Integrity Assessment (MAIA) device (iCare, Padova, Italy) at baseline and every 6 months for up to 24 months. All follow-up MP acquisitions were obtained using a ‘follow-up’ mode to allow registration to the baseline acquisition. MP testing was conducted in a dark room under pharmacologic pupil dilation while the contralateral eye was patched. MP testing was performed using a rectilinear 10 − 2 grid distribution (68 stimulus points; Goldman Size III (0.43° diameter)) centered on the anatomic fovea, with a 4 − 2 staircase threshold strategy and a 1° diameter red central fixation target. The average examination time was 8.8 minutes. All MP testing was performed prior to any imaging to prevent photoreceptor bleaching.

### Fundus Autofluorescence Imaging

Fundus autofluorescence (FAF) imaging was performed using the Spectralis HRA + OCT (Heidelberg Engineering, Heidelberg, Germany) at all study visits. In the high-speed mode, a 30° × 30° field centered on the fovea was imaged. FAF images consisted of 768 × 768 pixels.

### Junctional Zone MP Analysis

**Figure 1:**
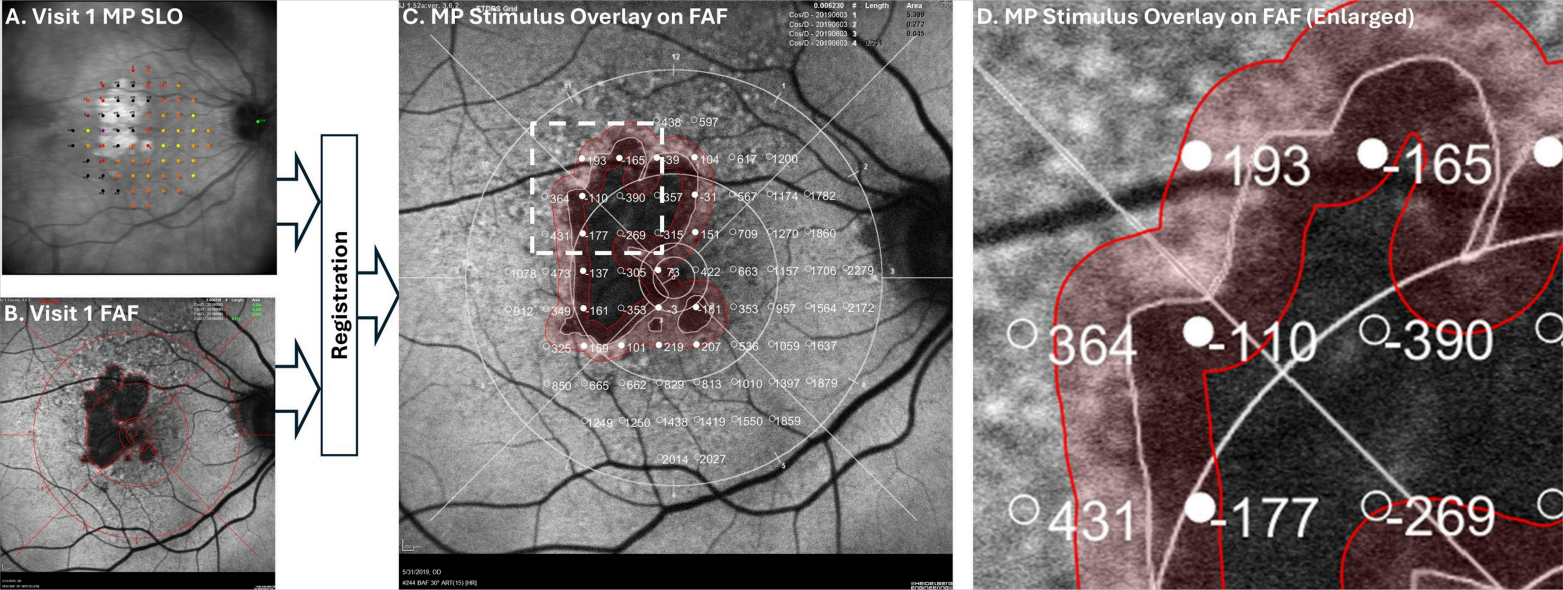
Workflow for longitudinal tracking of microperimetry (MP) sensitivities within the GA junctional zone. Baseline (visit 1) MP scanning laser ophthalmoscopy (SLO) images (panel A) are registered with baseline fundus autofluorescence (FAF) images (panel B), allowing the MP stimulus points to be overlaid on the FAF image (panels C, D). Each MP stimulus point is associated with a signed distance from the lesion margin, as defined by FAF tracing; distances, in micrometers, are shown next to each MP stimulus point. Positive and negative distances correspond to points outside and inside regions of atrophy, respectively. The junctional zone comprising all points within 250 μm of the lesion boundary is shaded red; those MP stimulus positions within the junctional zone have filled markers, and those outside the junctional zone have unfilled markers.

The workflow for GA junctional zone MP analysis, which follows the approach used by Hariri^9^, is presented in Fig. 1. In brief, using custom software, baseline MP images (sensitivity maps superimposed on their respective scanning laser ophthalmoscopy (SLO) fundus images) were registered to their corresponding baseline FAF images using fiducial markers manually positioned at corresponding bifurcations of the retinal vasculature. For registration, a similarity-type transformation (translation, rotation, and isotropic scaling) was used. The same transformation was then used to transform the MP stimuli coordinates into the FAF image coordinate frame. GA tracing was performed on the baseline FAF images, with the minimum lesion size defined as 0.05 mm^2 10,11^. With the GA tracings and MP measurements in the same coordinate frame (i.e., the FAF coordinate frame), the signed Euclidean (i.e., straight-line) distance from each MP stimulus point to the closest point on the baseline GA margin was computed. Negative and positive distances represent stimuli that lie inside and outside the areas of atrophy, respectively. A junctional zone, defined as all fundus positions within 250 μm of the GA margin (including regions both inside and outside the region of atrophy), was automatically generated. The position and width of the junctional zone used in this study matched that used for the MP analysis of the OAKS trial^12^.

### Statistical Analysis

Two graders (Grader 1 and Grader 2) performed registrations in a systematically stratified sampling of the 637 eyes from the OAKS study. The repeatability of the mean sensitivity and number of scotomatous points within the junctional zone were assessed using Bland–Altman analysis^13^, intraclass correlation coefficients (ICCs) and coefficients of repeatability (CoRs), also referred to as the smallest real difference^14^. To assess the repeatability of our MP analysis workflow without considering a particular junctional zone (e.g., ± 250 μm), we considered two measures, both of which incorporate all 68 stimulus points: (1) the repeatability of the signed-distance from each MP stimulus point to the GA margin (“stimulus-to-margin distance”; distances are negative for points within the GA lesion margin and positive for points outside the GA lesion margin), and (2) the distances between the coordinates of corresponding MP stimulus points when transformed by the two readers into the FAF coordinate frame (“stimulus coordinate difference”). The repeatability of the stimulus-to-margin distance was assessed using Bland-Altman analysis, as well as ICC and CoR. Following Taylor et al.^15^, linear mixed modeling was used to account for repeated measures. Stimulus coordinate differences were summarized with boxplots and descriptive statistics.

Note that all repeatability analyses assess only the repeatability of the MP *analysis*, which is determined by the repeatability of registering the MP SLO images to the FAF images. In particular, the repeatability analyses do not consider the repeatability of the MP acquisition, which have been reported by other authors^16^, or of the GA lesion tracing.

## Results

**Figure 2:**
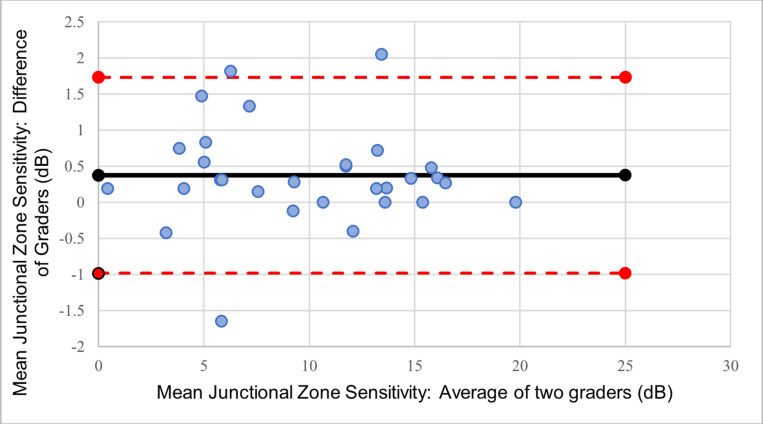
Bland-Altman plot of grader agreement for the mean sensitivity within the junctional zone between. (black line: bias; red dashed lines: upper and lower limits of agreement; blue dots: subjects)

**Figure 3:**
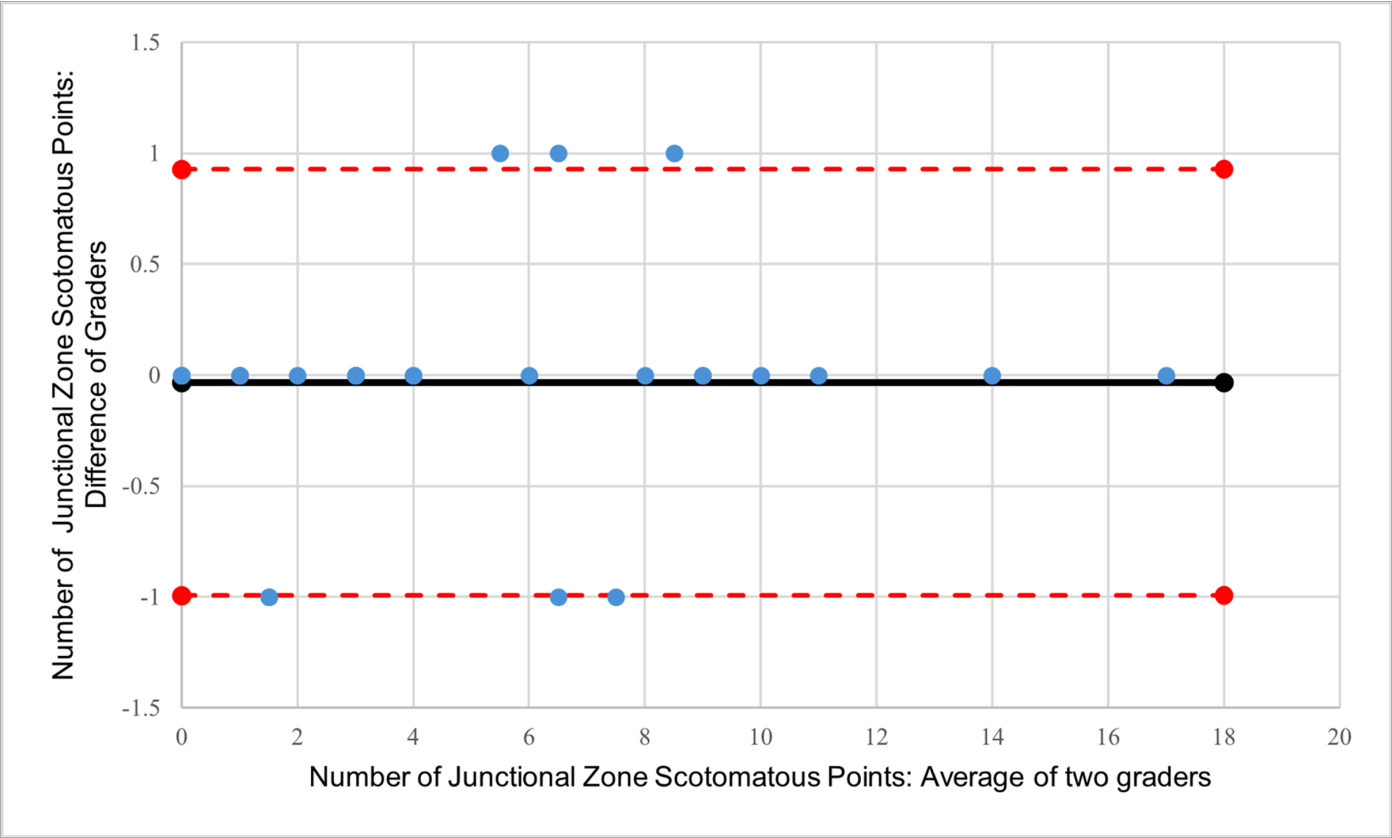
Bland-Altman plot of grader agreement for the total number of scotomatous points within the junctional zone. (black line: bias; ted-dashed lines: upper and lower limits of agreement; blue circles: subjects)

Images from thirty eyes (24 patients) were registered by two graders. Bland-Altman analysis of the mean sensitivity within the junctional zone showed a bias of 0.37 dB between the two graders, with 90% of the eyes within ± 1.96 SD (95% limit of agreement (LOA): −0.98 dB to 1.73 dB; Fig. 2). Bland-Altman analysis of total number of scotomatous points within the junctional zone showed a bias of 0.37 between the two graders, with 94% of the points within ± 1.96 SD (95% limit of agreement (LOA): −0.99 to 0.93; Fig. 3). The ICC and CoR for the mean junctional zone sensitivity were 0.994 and 0.349 dB, respectively. The ICC and CoR for the total number of scotomatous points within the junctional zone were 0.997 and 0.218, respectively.

**Figure 4:**
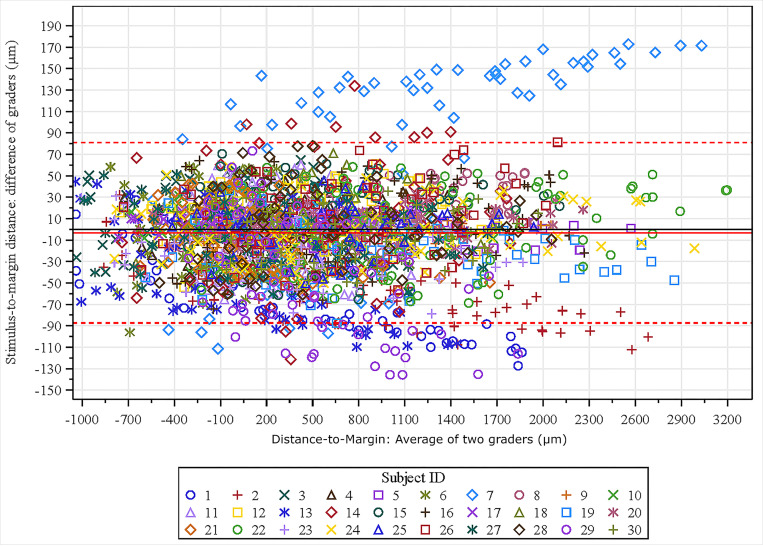
Bland-Altman plot of grader agreement of stimulus-to-margin distances across all measured stimulus points (68 per eye).

**Figure 5:**
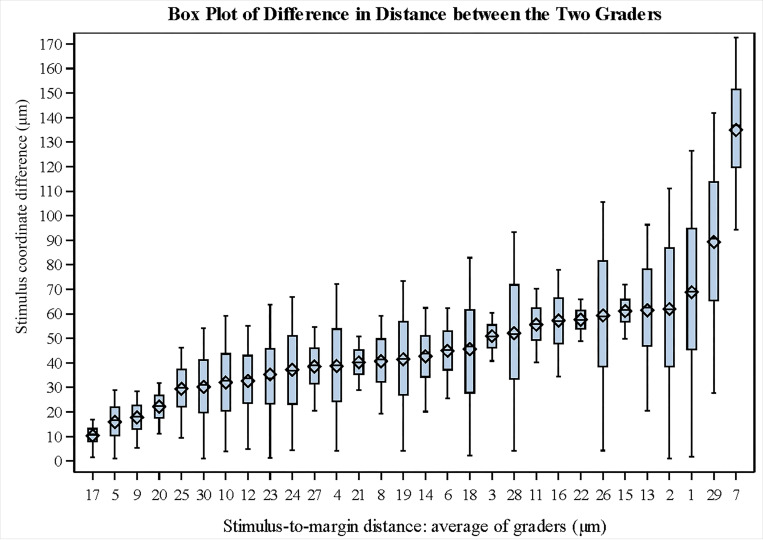
Box plot of the mean and standard deviation of the average stimulus coordinate differences over 68 stimulus points for each eye.

**Figure 6:**
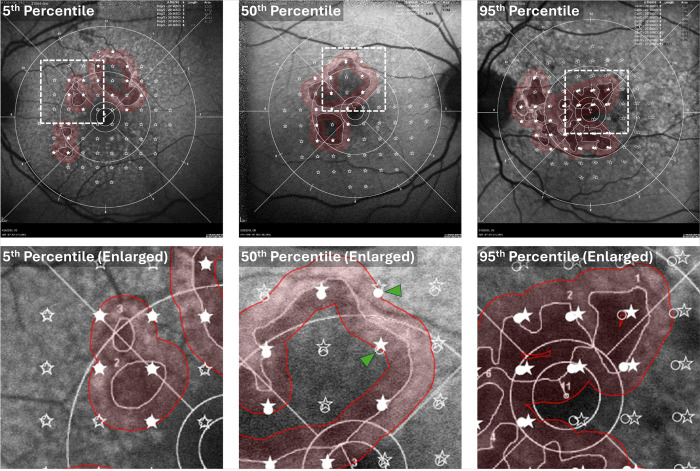
Representative Grader 1 and Grader 2 overlays of the microperimetry (MP) stimulus points on the fundus autofluorescence (FAF) images used for GA tracing. MP stimulus points are indicated by circle markers (Grader 1) and star markers (Grader 2). Filled and unfilled markers correspond to MP points inside and outside of the junctional zone, indicated by red shading, respectively. The images correspond to subjects having the 5^th^ percentile (Subject 5), 50^th^ percentile (subject 19), and 95^th^ percentile (Subject 29) mean stimulus coordinate differences (see Fig. 5). Enlargements of regions of interest specified by dashed boxes (top row) are shown in the bottom row. The green arrowheads indicate grader discrepancies in the assignment of points as being inside or outside of the junctional zone.

Bland-Altman analysis of the stimulus-to-margin distance, measured across all stimulus points (68 per eye), showed a mean shift of −3.24 μm between the two graders, with 94% of the points within ± 1.96 SD from the mean shift (95% limit of agreement (LOA): −87.35 μm to 80.87 μm; Fig. 4). Six eyes had larger variabilities (SD of difference > 50 μm) between the two graders and were outside the LOA bounds. The ICC and CoR of the stimulus-to-margin distances between the two graders were 0.969 and 99.85 μm, respectively. The stimulus coordinate differences are summarized in Fig. 5. The mean stimulus coordinate difference taken across stimulus points for all subjects was 46.87 ± 23.80 μm. Representative images of the registered MP in the FAF coordinate frame are shown in Fig. 6.

## Discussion

In the present study, we evaluated the repeatability of an MP analysis workflow that is compatible with clinical trial data (10 − 2 MP stimulus grid distribution and FAF imaging for GA tracing) and allows the visual function to be assessed in retinal areas that are most likely to be affected by GA growth—namely, the junctional zone comprised of the regions immediately surrounding the GA lesion margin. The repeatability of the method in the context of the mean junctional zone sensitivity and the number of scotomatous points within the junctional zone was excellent. Assessments of stimulus-to-margin distances and stimulus coordinate difference showed reader differences that were generally small compared to the junctional zone width, although there were some outlier cases (Figs. 4 and 5).

From a subjective review of grader registrations, we believe that the dominant cause of inter-grader discrepancies were likely due to the similarity-type image transformation not fully capturing the true deformation between the MP SLO and FAF images. To reduce inter-reader discrepancies in future studies, more general transformation types that better approximate the true deformation could be used. However, more general transformation types require more corresponding vessel bifurcations to be selected—in addition to increasing the grading time, selecting more vessel bifurcations can itself be error prone for images with less pronounced vasculature, and may even lead to larger discrepancies.

As in Hariri et al.,^9^ the MP workflow used in this study analyzes junctional zone sensitivities by transforming MP stimulus coordinates into the coordinate frame used for lesion tracing. After this transformation, the position of each MP stimulus point can be directly related to the GA lesion margin. An alternative approach to analyzing MP sensitivities in the junctional zone, proposed by Meleth et al.^17^ and recently used in the post hoc analysis of the Spectri and Chroma lampalizumab trials^18^, is to define the junctional zone using the set of scotomatous MP points. Such an approach has the advantage of simplicity (e.g., no image registration required). Furthermore, it can be performed using only MP data, making it particularly well suited to analyses in which GA tracing data are unavailable. However, for standard 10 − 2 MP stimulus grids, the 2-degree stimulus spacing suggests that a junctional zone derived using MP only is likely to be less accurate than a junctional zone derived directly from the registered GA tracing data.

Another approach to measuring junctional zone MP sensitivities is to use patient-tailored MP grids wherein the stimuli are distributed around the lesion margin^6,19^. An advantage of patient-tailored approaches is that MP measurements are not collected at fundus positions that are decoupled from lesion growth (e.g., regions of atrophy at baseline). Moreover, patient-tailored MP allows stimuli points to be distributed around lesions more uniformly and at higher-density within the junctional zone. A disadvantage is the requirement of customized, lesion-specific grids, which may complicate or be incompatible with current MP workflows and may become complex for certain lesion geometries (e.g., multifocal lesions). Moreover, the optimal grid parameters (e.g., stimulus density and junctional zone dimensions) are not *a priori* obvious. Indeed, one possible application of the MP approach used in the present study is in helping to design MP grid patterns for future studies using patient-specific MP.

An important limitation of our approach, particularly as applied to standard MP grids, is that the MP stimuli are relatively sparse and are randomly distributed relative to regions of atrophy. In addition to the possibility of missing smaller regions of functional impairment, there is a sparse and unequal sampling of the junctional zones, which we expect to increase variances when estimating treatment effects. One potential mitigation strategy is to model, or otherwise adjust for, the spatial distribution of stimulus points within the junctional zone. An alternate approach is to re-sample the MP measurements (e.g., via interpolation^20^) such that they uniformly tile the junctional zone. While these approaches also have limitations, we hope to explore these approaches in future studies.

## Conclusion

In this paper, we evaluate a microperimetry-based approach for assessing visual function changes in the GA junctional zone in a clinical trial context. The repeatability of the approach and its compatibility with standard MP acquisitions appear to make it well-suited to assessing the effects of GA therapeutics on visual function.
